# Dynamic change of blood profile in rat models with acute skin injury artificially infected with methicillin-resistant *Staphylococcus aureus*

**DOI:** 10.14202/vetworld.2021.2085-2090

**Published:** 2021-08-11

**Authors:** Yos Adi Prakoso, Nurul Hidayah, Chylen Setiyo Rini, Kurniasih Kurniasih

**Affiliations:** 1 Faculty of Veterinary Medicine, University of Wijaya Kusuma Surabaya, East Java, 60225, Indonesia; 2 Integrated Laboratory, Faculty of Health Sciences, University of Muhammadiyah Sidoarjo, East Java 61262, Indonesia; 3 Department of Pathology, Faculty of Veterinary Medicine, University of Gadjah Mada, Yogyakarta 55281, Indonesia

**Keywords:** blood profile, infection, methicillin-resistant *Staphylococcus aureus*, protein profile, wound

## Abstract

**Background and Aim:**

A wound is a common problem for humans and animals. The wound becomes more severe if it is infected by bacteria, such as methicillin-resistant *Staphylococcus aureus* (MRSA). The wound healing mechanism involves various factors, either in the local tissue or the bloodstream. However, the presentation of infected wound healing regarding its impacts on the dynamic change of blood profile is not clearly understood. This study aimed to explore the impacts of wound creation on the blood profile in rat models with and without being artificially infected by MRSA.

**Materials and Methods:**

Thirty male Sprague-Dawley rats (6 months old; weight, 300 g) were used as the model. They were divided into three groups: Without wound creation (C), wounded without infection (CW), and wounded and artificially infected by MRSA (CWI). Groups CW and CWI were shaved and induced with 4 mm two-round full-thickness biopsy on the back. Furthermore, group CWI was artificially infected by 105 colony-forming units of MRSA. The blood samples were collected through the tail vein from days 1 to 5. The blood parameters included blood profile, total plasma protein, C-reactive protein, CD4^+^, CD8^+^, CD4^+^/CD8^+^, and COX-2. The data were analyzed using the Statistical Package for the Social Sciences, version 16 (SPSS, IBM, Armonk, NY, USA).

**Results:**

The result showed that the presentation of a wound with and without MRSA infection significantly changed the total erythrocytes, leukocytes, neutrophils, lymphocytes, total plasma protein, C-reactive protein, and the subset of circulatory CD4^+^, CD8^+^, and COX-2 (p≤0.05). In addition, the wound infected with MRSA impacts the mean corpuscular volume (p≤0.05).

**Conclusion:**

Moreover, the presentation of the wound with and without MRSA infection induces dynamic changes on various blood profile parameters.

## Introduction

A wound is the disintegration of normal tissue. The wound is a common problem for humans and animals. Several types of wounds have been noted based on their appearances (e.g. closed and open wounds). The open wound is the most popular exploration aspect because it is related to esthetics [1]. Furthermore, the open wound can be easily infected by bacteria [2]. The most common one is methicillin-resistant Staphylococcus aureus (MRSA).

MRSA can produce various virulence factors that impair wound healing. These virulence factors promote MRSA by causing severe infection not only in a local tissue but also systemically [3]. MRSA can be transmitted into the bloodstream through an open wound. Further infection can make several histopathological changes in soft tissues and induce septicemia [4]. In wounds, the MRSA infection causes a more complex healing mechanism. The theory of wound healing shows intricate processes that included local cellular and immune responses [5], and may be it dramatically changes the involvement of systemic mechanisms. Furthermore, the early phase of infection in the wound caused by MRSA on changing of blood profile is not clearly understood.

The information regarding the impacts of MRSA in the wound on changing of blood profile is quite important because it provides supported data in wound management and its risk of infection, especially in an acute injury phase. Moreover, the change of blood profile could be used as the first indicator of systemic responses during infection of the host body. An acute injury phase becomes the major concern of this study because the correct handling and management of acute wounds prevent further local tissue damage and may cause systemic damage.

This study aimed to explore the impacts of wound creation on the blood profile in rat models with and without being artificially infected by MRSA.

## Materials and Methods

### Ethical approval

All animal experimentations in this study have been approved by the Ethical Clearance Committee, Faculty of Dental Medicine, University of Airlangga, East Java, Indonesia, with registration number: 312/HRECC.FOCM/VI/2019.

### Study period and location

This study was conducted from November 2019 to July 2020. The animal models were maintained at the Laboratory of Pharmacology, Faculty of Veterinary Medicine, University of Wijaya Kusuma Surabaya, East Java, Indonesia. The blood tests were measured at the Integrated Laboratory, Faculty of Health Sciences, University of Muhammadiyah Sidoarjo, East Java, Indonesia. The cell tube block was processed at the Department of Pathology, Faculty of Veterinary Medicine, University of Gadjah Mada, Yogyakarta, Indonesia.

### MRSA isolate

The MRSA isolate was obtained from the Laboratory of Bacteriology, Faculty of Health Sciences, University of Muhammadiyah Sidoarjo, Jawa Timur, Indonesia, with identification number 187/07/Lab. Bacteriology/TLM/2020. The isolate was enriched on Staphylococcus agar and transferred to the broth medium. Then, the isolate was incubated on an incubator at 37°C for 6 h until showing the turbidity of 0.5 McFarland.

### Animal models and research design

Thirty male Sprague-Dawley rats (6 months old; weight, 300 g) were used as a model. They were adapted for a week and maintained in an acrylic aquarium individually under standard laboratory conditions (standard laboratory animal feed and water *ad libitum* with 12/12 h light/dark). Furthermore, they were divided into three groups: Without wound creation (C), wounded without infection (CW), and wounded and artificially infected by MRSA (CWI). The rats were shaved on their back 1 day before wound creation. Groups CW and CWI were induced with 4 mm two-round full-thickness biopsy on the back using a biopsy puncher. The wounds of group CWI were artificially infected by 30 μL of 105 colony-forming units of MRSA.

### Blood and serum collection

The blood samples were collected through the tail vein from day 1 after induction until day 5. The blood specimen was separated into two parts. The first was stored inside the tube containing anticoagulant ethylenediaminetetraacetic acid. The second was stored inside the tube without anticoagulant and centrifuged until the serum was separated. Blood and serum were kept inside the fridge at 4°C until being tested.

### Blood and serum protein tests

The blood profile was analyzed using an automated hematology analyzer against several parameters, such as total erythrocytes, hemoglobin (Hb), packed cell volume (PCV), mean corpuscular volume (MCV), mean corpuscular Hb (MCH), MCH concentration (MCHC), total thrombocytes, leukocytes, neutrophils, and lymphocytes. Moreover, the serum was tested against total plasma protein and C-reactive protein (CRP). CRP was measured following the methods described in a previous study [6].

### Cell tube block

Blood was inserted into a plain capillary tube and rotated using a microhematocrit centrifuge at 12,000 rpm for 5 min. After centrifugation, the tube was broken in the plasma buffy coat using a diamond pen and soaked in 10% neutral buffer formalin for 24 h [7].

### Immunohistochemistry

Immunohistochemistry was performed using several primary antibodies, including CD4^+^(Novocastra, RTU-CD4-1F6, Cat. Number PA0427, USA) and COX-2 (Santa Cruz Biotechnology, COX-2 (D-12), Cat. Number sc-166475, USA). The tube was processed using xylene, graded alcohol (absolute, 90%, 80%, and 70%), and paraffin before immunohistochemistry staining. Furthermore, they were blocked using liquid paraffin. The block was sectioned using microtome at 3 μm of thickness and attached to the glass slide coated with poly-L-lysine. The CTB slides were stained with immunohistochemistry following the demonstrated procedure in a previous study [8].

### Statistical analysis

Normal and homogenous data were analyzed using the parametric test. In contrast, non-normal and nonhomogeneous data were analyzed using the non-parametric test. Following normality and homogeneity tests, the collected data in this study (e.g. erythrocytes, Hb, PCV, MCV, MCH, MCHC, thrombocytes, leukocytes, neutrophils, lymphocytes, total plasma protein, CRP, CD^+^, CD8^+^, CD4^+^/CD8^+^ ratio, and COX-2) were analyzed using two-way analysis of variance and *post hoc* tests. However, the data and percentage of the wound area were analyzed using an independent *t*-test. The statistical test was conducted using SPSS version 16. The significance level was considered at p≤0.05 level. The result was expressed as mean±standard deviation with a superscript for significantly different data.

## Results

This study showed a dramatic change regarding the number of circulatory erythrocytes in the CWI group compared with the control and CW groups (p≤0.05; Table-1). The decrease of erythrocytes in the CWI group began on day 2 after infection and continually decreased until day 5 during the study. The wound without MRSA infection in the CW group did not change the number of erythrocytes compared with the control during the observation period (p≥0.05; Table-1). The MCV in control and CW groups did not indicate a significant difference (p≥0.05). However, the MCV in the CWI group gradually increased along the observation time (p≤0.05; Table-1). In contrast, there were no significant differences between all groups regarding the value of Hb, PCV, MCH, and MCHC (p≥0.05; Table-1). Based on this finding, the wound with artificial MRSA infection promotes macrocytic normochromic anemia in rat models.

**Table-1:** Profile of erythrocytes, Hb, PCV, MCV, MCH, and MCHC in rat models with acute skin injury infected with methicillin-resistant *Staphylococcus aureus*.

Parameter	Group	Days (mean±standard deviation)				

		1	2	3	4	5
Erythrocytes (10^6^ cells/mm^3^)	C	5.59±0.03*	5.63±0.03*	5.59±0.03*	5.62±0.03*	5.59±0.00*
	CW	5.58±0.06*	5.47±0.04*	5.50±0.02*	5.60±0.01*	5.62±0.01*
	CWI	5.48±0.06**	5.43±0.04**	5.38±0.07**	5.33±0.06**	5.25±0.20**
Hb (g/dL)	C	14.84±0.39	14.46±0.54	14.68±0.33	14.68±0.26	14.44±0.40
	CW	13.97±0.15	13.63±0.23	14.08±0.24	14.23±0.37	14.41±0.37
	CWI	14.13±0.57	13.76±0.48	14.12±0.83	13.70±0.47	13.84±0.25
PCV (%)	C	41.23±0.61	40.88±0.45	41.60±1.38	41.20±0.84	41.24±0.74
	CW	41.06±0.56	40.74±0.55	41.68±1.20	41.12±0.72	41.12±0.38
	CWI	41.08±0.60	41.08±1.02	41.38±0.56	41.16±0.47	41.36±0.58
MCV (fL)	C	73.68±1.46*	72.64±1.17*	74.37±2.78*	73.31±1.48*	73.66±1.31*
	CW	73.51±1.32*	74.45±1.05*	75.75±2.27*	73.37±1.31*	73.11±0.59*
	CWI	74.97±1.64**	75.63±2.21**	76.90±1.81**	77.16±0.76**	78.86±2.96**
MCH (Pg)	C	26.52±0.76	25.52±0.92	26.24±0.57	26.12±0.50	25.79±0.74
	CW	25.02±0.20	24.91±0.47	25.59±0.51	25.39±0.68	25.62±0.64
	CWI	25.79±0.90	25.32±0.71	26.25±1.76	25.68±1.00	26.42±1.46
MCHC (%)	C	36.00±1.19	35.37±1.46	35.32±1.17	35.65±1.35	35.02±1.05
	CW	34.04±0.64	33.46±0.34	33.79±0.85	34.62±1.37	35.04±0.91
	CWI	34.41±1.38	33.52±1.85	34.14±2.10	33.29±1.42	33.48±0.89

* Different superscript indicates significant differences (p≤0.05). Hb=Hemoglobin, PCV=Packed cell volume, MCV=Mean corpuscular volume, MCH=Mean corpuscular hemoglobin, MCHC=Mean corpuscular hemoglobin concentration

Further analysis demonstrated that the coagulant factor (e.g. thrombocytes) did not show a significant difference between all groups (p≥0.05; Table-2). Surprisingly, the local injury in the rat skin significantly changed the number of circulatory leukocytes, neutrophils, and lymphocytes in the CW and CWI groups compared with the control group (p≤0.05; Table-2). The increase of neutrophils and lymphocytes in the CW and CWI groups occurred from day 1 after induction until day 5. The increasing value of neutrophils and lymphocytes in the CWI group was greater than the CW group (p≤0.05; Table-2).

**Table-2:** Profile of thrombocytes, leukocytes, neutrophils, and lymphocytes in rat models with acute skin injury infected with methicillin-resistant *Staphylococcus aureus*.

Parameter	Group	Days (mean±standard deviation)				

		1	2	3	4	5
Thrombocytes (10^5^ cells/mm^3^)	C	4.41±0.15	4.31±0.07	4.22±0.08	4.22±0.08	4.14±0.08
	CW	4.30±0.21	4.29±0.15	4.22±0.08	4.20±0.07	4.14±0.08
	CWI	4.22±0.19	4.10±0.14	4.04±0.11	4.00±0.25	4.00±0.35
Leukocytes (10^3^ cells/mm^3^)	C	6.28±0.35*	6.30±0.31*	6.31±0.35*	6.14±0.28*	6.16±0.21*
	CW	7.42±0.68**	7.48±0.84**	8.98±0.18**	9.00±0.18**	8.97±0.47**
	CWI	9.47±2.21***	10.07±1.82***	10.21±2.26***	11.28±0.68***	11.44±1.15***
Neutrophil (10^3^ cells/mm^3^)	C	1.74±0.18*	1.65±0.18*	1.62±0.19*	1.58±0.13*	1.41±0.12*
	CW	1.98±0.29**	1,86±0.20**	2.51±0.10**	2.43±0.21**	2.30±0.31**
	CWI	2.38±0.56***	2.73±0.51***	2.75±0.56***	3.13±0.16***	2.96±0.25***
Lymphocytes (10^6^ cells/mm^3^)	C	3.90±0.22*	4.02±0.30*	4.04±0.24*	3.94±0.22*	4.13±0.27*
	CW	4.70±0.38**	4.86±0.59**	5.57±0.09**	5.67±0.15**	5.77±0.36**
	CWI	6.13±1.52***	6.33±1.16***	6.43±1.52***	7.01±0.48***	7.33±0.88***

* Different superscript indicates significant differences (p≤0.05).

A similar result was demonstrated by total plasma protein and CRP in the CW and CWI groups compared with the control (p≤0.05; Table-3). An increase in total plasma protein and CRP was noted on day 1 after injury. However, a different pattern for these parameters was noted in the next few days. The total plasma protein in the CW group increased until day 2 after injury and gradually decreased on day 3 until the final day. Moreover, the CRP of the CW group increased until day 3 and decreased on days 4-5. However, the total plasma protein and CRP in the CWI group continuously increased from days 1 to 5 without showing any decreasing pattern (Table-3).

**Table-3:** Profile of total protein plasma and C-reactive protein in rat models with acute skin injury infected with methicillin-resistant *Staphylococcus aureus.*

Parameter	Group	Days (mean±standard deviation)				

		1	2	3	4	5
Protein plasma (g/dL)	C	6.60±0.20*	6.60±0.37*	6.48±0.25*	6.50±0.18*	6.50±0.18*
	CW	8.76±0.32**	9.34±0.11**	8.86±0.42**	7.50±0.12**	6.44±0.36**
	CWI	9.32±0.37***	10.32±0.59***	10.80±1.28***	11.88±0.77***	12.84±1.48***
CRP (mg/dL)	C	34.20±1.64*	32.60±1.67*	32.40±1.51*	32.40±2.07*	32.80±1.92*
	CW	52.60±5.02**	69.60±5.50**	84.20±3.27**	67.20±6.30**	41.40±8.44**
	CWI	56.60±3.78***	66.80±21.58***	83.00±3.16***	86.00±5.95***	92.20±4.86***

* Different superscript indicates significant differences (p≤0.05).

The leukocyte increase was followed by the increase of circulatory CD4^+^, CD8^+^, and COX-2 in the CW and CWI groups (p≤0.05; Table-4) but not the CD4^+^/CD8^+^ ratio (p≥0.05; Table-4). The CD4^+^ increase in the CW group concomitantly occurred with the CD8^+^ increase. However, the CD4^+^ increase in the CWI group was slower than the CD8^+^ increase. It impacted the CD4^+^/CD8 ratio in this study. The stimulation of wound creation increased the number of circulatory COX-2 in this study. Furthermore, the increase of COX-2 in the CW group reached the highest peak on day 2 and gradually decreased the next day. However, the MRSA infection in the CWI group induced the COX-2 expression to continuously become higher until the final day (p≤0.05; Table-4). Based on the finding, the changing of the several aforementioned parameters is related to the wound presentation in the rat skin either with or without MRSA infection (p≤0.05; Table-5).

**Table-4:** Profile of CD4+, CD8+, ratio CD4+/CD8+, and COX-2 in rat models with acute skin injury infected with methicillin-resistant *Staphylococcus aureus.*

Parameter	Group	Days (mean±standard deviation)				

		1	2	3	4	5
CD4+ (%)	C	2.86±0.50*	2.86±0.56*	2.56±0.61*	2.46±0.50*	2.64±0.95*
	CW	2.99±0.28**	3.87±0.71**	5.64±0.44**	6.04±0.41**	5.74±0.51**
	CWI	2.89±0.36***	3.13±0.53***	2.69±0.52***	3.58±0.34***	3.59±0.62***
CD8+ (%)	C	1.70±0.34*	1.64±0.51*	1.46±0.40*	1.44±0.47*	1.64±0.33*
	CW	1.97±0.16**	1.89±0.42**	3.03±0.83**	3.07±0.57**	4.30±0.36**
	CWI	2.80±0.56***	2.45±1.05***	3.58±0.29***	3.70±0.17***	3.61±0.51***
Ratio CD4+/CD8+	C	1.78±0.67	1.81±0.29	1.81±0.49	1.83±0.61	1.66±0.76
	CW	1.53±0.23	2.18±0.89	1.97±0.50	2.02±0.39	1.34±0.18
	CWI	1.05±0.13	1.53±0.75	0.74±0.12	0.96±0.05	1.02±0.31
COX-2 (%)	C	3.32±0.45*	3.34±0.34*	3.25±0.24*	3.46±0.31*	3.18±0.34*
	CW	5.32±0.49**	5.42±0.97**	4.04±0.64**	3.52±0.54**	3.07±0.16**
	CWI	6.48±1.00***	6.41±1.96***	6.87±0.67***	6.60±1.20***	7.16±0.72***

* Different superscript indicates significant differences (p≤0.05).

**Table-5:** Comparison of wound area and percentage wound area in rats models with acute skin injury infected with methicillin-resistant *Staphylococcus aureus*.

Parameter	Group	Days (mean±standard deviation)				

		1	2	3	4	5
Wound area (mm^2^)	C	NT	NT	NT	NT	NT
	CW	16.00±0**	14.31±0.69**	10.95±1.48**	6.61±1.20**	4.09±1.36**
	CWI	16.00±0**	14.17±2.35**	14.39±1.38***	14.13±1.66***	13.34±0.75***
Percent wound area (%)	C	NT	NT	NT	NT	NT
	CW	100.00±0**	89.46±4.35**	68.45±9.25**	41.37±7.55**	25.59±8.50**
	CWI	100.00±0**	88.58±14.71**	89.94±8.68***	88.33±10.43***	83.41±4.73***

*Different superscript indicates significant differences (p≤0.05), NT=Not tested due to the control group did not receive any wound creation.

## Discussion

Blood profile is one indicator to find out disease pathogenesis and prognosis. The common disease type that contributes to changing of blood profile is the infectious disease that is caused either by a virus or bacteria. The most eminent bacteria that caused the infection in humans are MRSA. MRSA is the leading highest cause of infection worldwide [9]. MRSA can generate infection through several exposure routes, especially from direct contact with the tools contaminated with MRSA. Direct contact with low hygiene practices increases the prevalence of MRSA infection. It is proved by the high number of detected MRSA using multilocus sequence typing in patients with hospitalization history [10]. Moreover, a secondary or primary infection caused by MRSA in comorbid patients can lead to more severe clinical signs if it transmits systemically to the bloodstream, including hematological profile changing. The hematological change in an infected patient with MRSA is being suspected to occur following the transmission of this bug through the open wound.

This study proved that artificial MRSA infection on acute injury causes anemia macrocytic normochromic in a mouse model. The anemia macrocytic normochromic is caused by the increasing volume of erythrocytes related to systemic infection [11] and destruction of the endothelial cells [12]. Furthermore, it causes erythrolysis that promotes the increased need for O_2_ and nutrients within the tissue. These mechanisms depress the bone marrow to release the reticulocytes with less capacity of Hb within the bloodstream [13]. Unfortunately, MRSA isolation from a blood specimen was not conducted in this study. Thus, the presence of systemic infection due to the artificial MRSA infection in the acute wound in this study remains presumptive.

Nevertheless, this study found that a dramatic change exists regarding the number of circulatory leukocytes, neutrophils, and lymphocytes following the artificial MRSA infection in an acute wound. The increase of those parameters directly occurs on day 1. It proved that the presence of MRSA in wound acts as the chemoattractant for circulatory leukocytes, neutrophils, and lymphocytes. The increase of leukocytes potentially affects cytokine synthesis [14]. Moreover, the neutrophil is an agent in both circulation and tissue in controlling and destroying an infectious agent and mediates the inflammatory responses and tissue pathogen [15]. Within the tissue and circulation, the neutrophils can form pseudopodia resembling a net that is called neutrophil extracellular traps [16].

Another blood parameter that increased was lymphocytes. There are two types of lymphocytes (i.e. T and B lymphocytes). Both of them have a significant role in the regulation of healing and controlling the pathogen. In this study, the T lymphocytes become the main indicator. The increase of T helper (CD4^+^) in the CW group expressed that the healing mechanism occurs faster than the CWI group. It is supported by a previous study that demonstrates that the increase of CD4^+^ rather than CD8^+^ could be used as the marker for better healing processes [17,18]. Furthermore, the representation of COX-2 in the CWI group was greater than the CW group, indicating that the oxidative stress in the group with infection was more severe than in the group without infection. It relates to the glycation mechanism in an infected group that gets worse following the observation time [19].

The involvement of leukocytes, neutrophils, lymphocytes, COX-2, and CD8^+^ that increase in the infected group impacts the higher synthesis of CRP and total plasma protein compared with the control and CW groups. These mechanisms occur due to leukocytes, neutrophils, lymphocytes, COX-2, and CD8^+^ acting as the mediator of inflammation that induces the synthesis of the acute-phase protein [20]. The increased value of leukocytes, neutrophils, lymphocytes, COX-2, CD8^+^, plasma protein, and CRP in an acute injury with MRSA infection indicates that the body goes through a strenuous mechanism compared to the non-infected and control groups. If it continuously occurs and/or happens to a comorbid patient, it will impact the index of erythrocytes, including anemia macrocytic normochromic or may be with more severe pathogenesis and prognosis [21].

This study proves that MRSA can be transmitted through the acute wound and change the blood profile in rat models. It is related to the wider presentation and percent of the wound area in the infected group than in the non-infected group. The wider wound area and percent of wound area in the CWI group enable MRSA transmission through the peripheral blood vessel surrounding the wound area that eventually changes the blood profile parameters.

## Conclusion

The artificial MRSA infection through the acute injury promotes the changing of blood profile such as total erythrocytes, leukocytes, neutrophils, lymphocytes, total plasma protein, CRP, the subset of circulatory CD4^+^, CD8^+^, and COX-2 and leads to anemia macrocytic normochromic. The change of blood profile in the infected group occurs more severely compared with the non-infected group that is similar to the presentation of the wound area in rat models, while the previous study did not mention this information. Further study with a deeper parameter (e.g. bacterial culture and gene expression from blood specimen) is needed to provide the complex pathogenesis mechanism of this bug.

## Authors’ Contributions

YAP, CSR, and KK: Designed the research methodology and conducted the study. YAP and CSR: Prepared the MRSA isolate. NH and YAP: Write the concept of the manuscript. All authors read and approved the final manuscript.
